# Disorders of Iron Metabolism: A “Sharp Edge” of Deoxynivalenol-Induced Hepatotoxicity

**DOI:** 10.3390/metabo15030165

**Published:** 2025-03-01

**Authors:** Haoyue Guan, Yujing Cui, Zixuan Hua, Youtian Deng, Huidan Deng, Junliang Deng

**Affiliations:** Key Laboratory of Animal Disease and Human Health of Sichuan Province, Sichuan Agricultural University, Chengdu 611130, China; guanhaoyue08@163.com (H.G.); yjchoi@163.com (Y.C.); 13708235219@163.com (Z.H.); idyt@outlook.com (Y.D.); denghuidan@sicau.edu.cn (H.D.)

**Keywords:** iron metabolism, deoxynivalenol, liver, ferroptosis, Nrf2

## Abstract

Background/Objectives: Deoxynivalenol (DON), known as vomitoxin, is one of the most common mycotoxins produced by *Fusarium graminearum*, with high detection rates in feed worldwide. Ferroptosis is a novel mode of cell death characterized by lipid peroxidation and the accumulation of reactive oxygen species. Although it has been demonstrated that DON can induce ferroptosis in the liver, the specific mechanisms and pathways are still unknown. The aim of this experiment was to investigate that DON can induce iron metabolism disorders in the livers of mice, thereby triggering ferroptosis and causing toxic damage to the liver. Methods: Male C57 mice were treated with DON at a 5 mg/kg BW concentration as an in vivo model. After sampling, organ coefficient monitoring, liver function test, histopathological analysis, liver Fe^2+^ content test, and oxidative stress-related indexes were performed. The mRNA and protein expression of Nrf2 and its downstream genes were also detected using a series of methods including quantitative real-time PCR, immunofluorescence double-labeling, and Western blotting analysis. Results: DON can cause damage to the liver of a mouse. Specifically, we found that mouse livers in the DON group exhibited pathological damage in cell necrosis, inflammatory infiltration, cytoplasmic vacuolization, elevated relative liver weight, and significant changes in liver function indexes. Meanwhile, the substantial reduction in the levels of glutathione (GSH), catalase (CAT), superoxide dismutase (SOD), and total antioxidant capacity (T-AOC) in the DON group indicated that DON also caused oxidative stress in the liver. Notably, DON exposure increased the levels of Fe^2+^ and Malondialdehyde (MDA) in the liver, which provides strong evidence for the occurrence of iron metabolism and ferroptosis disorders. Most importantly, mRNA and protein expression of Nrf2, an important pathway for iron metabolism and ferroptosis, along with its downstream genes, heme oxygenase (HO-1), quinone oxidoreductase (NQO1), glutathione peroxidase (GPX4), and solute carrier gene (SLC7a11), were significantly inhibited in the DON group. Conclusions: Based on our results, the Nrf2 pathway is closely associated with DON-induced iron metabolism disorders and ferroptosis in mouse livers, suggesting that maintaining hepatic iron homeostasis and activating the Nrf2 pathway may be a potential target for mitigating DON hepatotoxicity in the future.

## 1. Introduction

DON is one of the most common mycotoxins produced by *Fusarium graminearum* and is detected at high rates in feed and feed ingredients worldwide [1]. The contamination of food and feed food by DON is a serious problem in many countries worldwide, as DON not only causes economic losses but also poses a serious threat to human health and food safety [2]. Furthermore, DON is also known as the “vomitoxin”, and it produces toxic effects in humans and animals, causing diarrhea, anorexia, vomiting, growth retardation, and immunotoxicity [3,4,5], and in severe cases, teratogenicity [6] and carcinogenicity [7]. DON can be absorbed quickly and efficiently in the body and distributed to various organs and tissues such as the stomach and intestines, liver, kidneys, and brain after being ingested by animals, with the highest content in the liver [8]. As a primary target organ, the liver is particularly vulnerable to the toxic effects of DON. When the dose or residual accumulation of DON exceeds the metabolizing capacity of the liver, it can lead to acute or chronic liver injury and growth retardation in animals [9]. In previous studies, it was found that the liver can convert DON to the less toxic DON-glucuronide (DON-GlcA) via UDP-Glycosyltransferase (UGT) after DON enters the body of humans and animals, thus reducing the harmful effects of DON [10]. Clearly, it can be seen that the liver plays an essential protective role in the toxicity and damage caused to the organism by DON. Therefore, research on the molecular mechanism of DON toxicity to the liver and the identification of the critical pathways of DON-induced liver injury can provide new insights for the prevention, control, and treatment of DON-induced diseases in humans and animals.

Ferroptosis is a novel mode of iron-dependent cell death characterized by the accumulation of lipid reactive oxygen species, leading to cell death in a distinct morphology and function compared with standard cell death modes such as necrosis, apoptosis, and autophagy [11]. It has been demonstrated in previous studies that DON-induced intestinal injury in pigs [12], testicular injury in mice [13], and damage to the liver as well as hepatocytes in mice [14,15] are directly associated with ferroptosis, and that the liver plays a crucial role in ferroptosis [16]. Although many researchers in recent years have successively demonstrated that ferroptosis occurs during DON-induced liver injury, the specific mechanism of DON-induced ferroptosis in the liver is still unclear. The primary roles of the Nrf2 pathway are erythropoiesis and the regulation of cellular redox and cellular stress pathways [17]. With the increasing research on ferroptosis, many scholars have found that the Nrf2 pathway is also a key factor in regulating ferroptosis. GPX4, SLC7a11, HO-1, and many other proteins and enzymes responsible for preventing the triggering of ferroptosis via lipid peroxidation are downstream targets of Nrf2 [18]. In studies of DON toxicity and its association with ferroptosis, the research focus has also gradually favored the Nrf2 pathway. Recently, it has also been found that DON can induce ferroptosis in mouse testes by modulating the Nrf2/system Xc/GPX4 system [13], and it has also been found that selenomethionine reduces the damage caused by DON to the liver through the Nrf2/PPARγ-GPX4-ferroptosis pathway in mice [15]. Therefore, the study of Nrf2 and its downstream pathway is essential in DON-induced ferroptosis occurring in the liver.

As an environmental pollutant, DON seriously threatens human and animal health and causes severe damage to the liver, an essential metabolic organ. The liver is susceptible to oxidative damage, and excessive iron accumulation is a significant feature of most liver diseases. As a new cell death mode, ferroptosis plays a vital role in liver diseases and provides a new way of thinking about and treating many diseases. Currently, there are fewer studies based on this aspect of ferroptosis on DON-induced liver injury. Therefore, the present experiments were carried out to investigate the mechanism of DON-induced ferroptosis in mouse livers using the key pathway of ferroptosis, Nrf2, as a target. This will provide new ideas for further research on the mechanism of DON-induced liver injury in animals and how to carry out targeted therapy.

## 2. Materials and Methods

### 2.1. Animal Grouping and Sampling

The test animals were 24 male C57 mice of SPF grade (SiPeiFu, Beijing, China), grown at 3 weeks of age. The mice were allowed to feed and drink freely, and the bedding was changed every 2 d. Feeding temperature was strictly controlled at 18–22 °C, humidity was maintained between 50% and 60%, and a 12/12 h light/dark cycle was performed daily. The mice were acclimatized for 1 week before the test. At the beginning of the test, the mice were simply randomized into two groups, and each group was fed the mouse basal diet. Test group 1 was the control group, and test group 2 was the DON group. Mice in the DON group were administered DON (purity > 99%) (ELPIZO, Shanghai, China) at a concentration of 5 mg/kg BW by gavage at 9:00 a.m. every day, while the control group was administered an equal amount of saline for 21 d. To ensure the accuracy of the test results, as well as to improve the success rate of modeling, the mice were fasted for 4 h before sampling on day 21. The mice were anesthetized with isoflurane before orbital blood sampling for biochemical parameter analysis (100–150 μL of whole blood was taken and added to blood collection tubes pre-flushed with sodium heparin. Plasma was collected by centrifugation (1500× *g* at 4 °C for 15 min) and subsequently stored at −80 °C until further analysis). Mice were euthanized via cervical dislocation after blood collection. After dissection, a portion of the mouse liver tissue was placed in fixative for observation of hematoxylin and eosin (HE) staining, and the rest was immediately stored in liquid nitrogen for further determination.

### 2.2. Liver Function Assessment

Hematological parameters were determined using a fully automated biochemical analyzer (BS360S, mindray, Shenzhen, China) (ALB, albumin; ALP, alkaline phosphatase; ALT, alanine aminotransferase; AST, aspartate aminotransferase; CHO, total cholesterol; GLB, globulin; T-bil, total bilirubin).

### 2.3. Liver Histomorphology Analysis

Fresh mouse liver tissues were first fixed with a fixative (4% paraformaldehyde) for 24 h. Then, they were dehydrated, embedded, sectioned, stained with HE to enhance the tissue contrast, and finally, microscopically examined using a microscope. The images were acquired and analyzed.

### 2.4. Liver Fe^2+^ Content

The Fe^2+^ content of the liver tissues of the two groups of mice was determined according to the kit instructions (Elabscience, Wuhan, China). The OD values were determined at 593 nm using an enzyme marker and finally calculated according to the formula provided in the instructions.

### 2.5. Measurement of Oxidative Stress and Antioxidant Indicators

Liver tissue proteins were measured using a bicinchoninic acid (BCA) protein assay kit (Beyotime Institute of Biotechnology, Shanghai, China). Several critical oxidative stress and antioxidant indices were determined in the liver tissues of mice from both groups in this experiment using kits. Superoxide dismutase (SOD, Nanjing Jianjian Bioengineering Research Institute, Nanjing, China); total antioxidant capacity (T-AOC, Nanjing Jianjian Bioengineering Research Institute, Nanjing, China) and malondialdehyde (MDA, Nanjing Jianjian Bioengineering Research Institute, Nanjing, China); reduced glutathione (GSH, Nanjing Jianjian Bioengineering Research Institute, Nanjing, China); Certified Accounting Technician (CAT, Solarbio, Beijing, China). The OD value of each index was determined by applying a 752 N UV–visible spectrophotometer, and the results were calculated according to the instructions.

### 2.6. Quantitative Real-Time Polymerase Chain Reaction (qRT-PCR)

Total RNA was extracted from mouse livers using a Triazole reagent and an RNA extraction kit (Accurate, Changsha, China). After extraction, total RNA was reverse-transcribed into cDNA using the Evo M-MLV Reverse Transcription Premix Kit (Accurate, Changsha, China) according to the manufacturer’s instructions. Relative mRNA expression of target genes was normalized relative to the housekeeping gene β-actin, and gene expression was calculated using the 2^−ΔΔCT^ formula. Primer sequences for each gene are shown in Table S1.

### 2.7. Immunofluorescence Double Staining

The liver tissues were embedded and fixed in sections, and the sections were sequentially placed in xylene I for 15 min, xylene II for 15 min, anhydrous ethanol I for 5 min, anhydrous ethanol II for 5 min, 85% alcohol for 5 min, 75% alcohol for 5 min, and then washed with distilled water. The sections were closed with goat serum after antigenic repair with EDTA antigenic repair buffer. Primary antibodies and corresponding HRP-labeled secondary antibodies were added sequentially at the following dilutions: Nrf2 (1:100, Bioss, Beijing, China), SLC7a11 (1:150, Bioss, Beijing, China), NQO1 (1:100, Bioss, Beijing, China), HO-1 (1:250, Abcam, Shanghai, China), GPX-4 (1:100, Proteintech, Wuhan, China), COX-2 (1:500, Abcam, Shanghai, China). Finally, the nuclei were re-stained with DAPI, and the images were observed and captured under a fluorescence microscope (Leica, DM6000B, Wetzlar, Germany).

### 2.8. Western Blotting Analysis

Total protein was isolated from liver tissue using radioimmunoprecipitation assay (RIPA) buffer (Beyotime, Shanghai, China). The protein concentration in the extracted samples was determined using a BCA protein assay kit (Nanjing Jianjian Bioengineering Research Institute, Nanjing, China). Proteins were then blotted using phenylmethylsulfonyl fluoride (PMSF) (100 mmol/L). Proteins were separated from sodium dodecyl sulfate–polyacrylamide gel electrophoresis (SDS-PAGE) gels at 12% or 10% concentration by SDS–polyacrylamide gel electrophoresis and transferred to nitrocellulose (NC) membranes at a constant current of 200 mA. The transferred membranes were placed in an incubator with TBST and closed with 5% bovine serum albumin (BSA) for 2 h. The membranes were incubated with a membrane against β-actin (1:1500, Abcam, Shanghai, China), Nrf2 (1:1000, CST, Boston, USA), SLC7a11 (1:1000, Abcam, Shanghai, China), NQO1 (1:10,000, Abcam, Shanghai, China), HO1 (1:1000, CST, Boston, USA), GPX-4 (1:1000, CST, MA, USA), COX-2 (1:1000, Abcam, Shanghai, China) and were incubated overnight with diluted primary antibodies. The membranes incubated with primary antibodies were washed with TBST 3 times, each for 5 min. Subsequently, according to the dosage, the HRP-labeled secondary antibody (goat anti-rabbit HRP-labeled secondary antibody, beyotime, Shanghai, China) was diluted according to 1:1000, and the membranes were incubate with the membrane for 1 h at 37 °C. The membranes were washed with TBST 3 times, each time for 5 min. Finally, the bands were detected with an ECL kit (Biosharp, Beijing, China) on a chemiluminescent imaging system (Azure Biosystems C300, Azure Biosystems Inc., Dublin, CA, USA).

### 2.9. Statistical Analyses

ImageJV1.8.0 was used to examine protein blotting with immunofluorescence results. Images were acquired with GraphPad Prism 10.0 (GraphPad software). Differences between groups were assessed using Student’s *t*-test or one-way ANOVA, while Pearson correlation analysis was used to assess correlation. Differences were considered significant at *p <* 0.05 (*, *p* < 0.05; **, *p* < 0.01; and ***, *p* < 0.001). Spearman’s correlation coefficient (*p* < 0.05) was calculated using the “Hmisc” R package.

## 3. Results

### 3.1. DON-Induced Liver Injury in Mice

**Figure 1:**
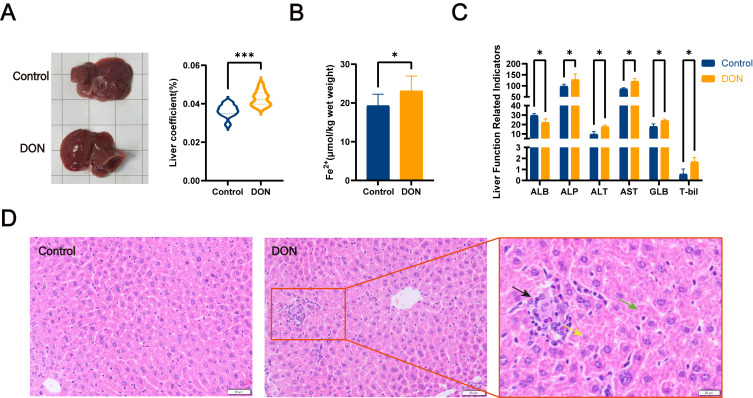
Assessment of liver injury in mice. (**A**) Visceral coefficients of the control and DON groups (*n* = 12, values represent ± SEMs). (**B**) Detection of Fe^2+^ content in mouse liver tissue (*n* = 12, values represent ± SEMs). (**C**) Assessment of mouse liver function-related indexes, including ALB, ALP, ALT, AST, GLB, and T-bil (*n* = 12, values represent ± SEMs). (**D**) Histopathological analysis of H&E-stained mouse livers (magnification ×20, bar = 50 μm). Black arrows are inflammatory cell infiltration, yellow arrows are cell necrosis, and green arrows are cytoplasmic vacuolization. No obvious lesions were seen in the control group. * (*p* < 0.05) vs. Control, *** (*p* < 0.001) vs. Control.

In order to investigate the effects of DON on the mouse liver, relative liver weight, liver iron ion content, and histopathology were monitored during the experiment. As shown in Figure 1A, the organ coefficient of the liver was significantly increased in the DON group (*p <* 0.01). In addition, in the liver tissue Fe^2+^ content assay (Figure 1B), the Fe^2+^ content was significantly higher in the DON group (*p* < 0.05). Meanwhile, we found in our liver function-related indexes assay (Figure 1C) that DON exposure caused a decrease in liver function in mice, as evidenced by a significant reduction in ALB values as well as a significant increase in ALP, ALT, AST, GLB, and T-bil values in the DON group compared with the control group (*p* < 0.05). In our histological analysis (Figure 1D), the DON group also had significant pathological changes compared with the control group. DON could trigger inflammatory cell infiltration (black arrows), hepatocellular necrosis (yellow arrows), and cytoplasmic vacuolization (green arrows) in the livers of mice.

### 3.2. DON-Induced Changes in Liver Oxidative Stress-Related Indices in Mice

**Figure 2:**
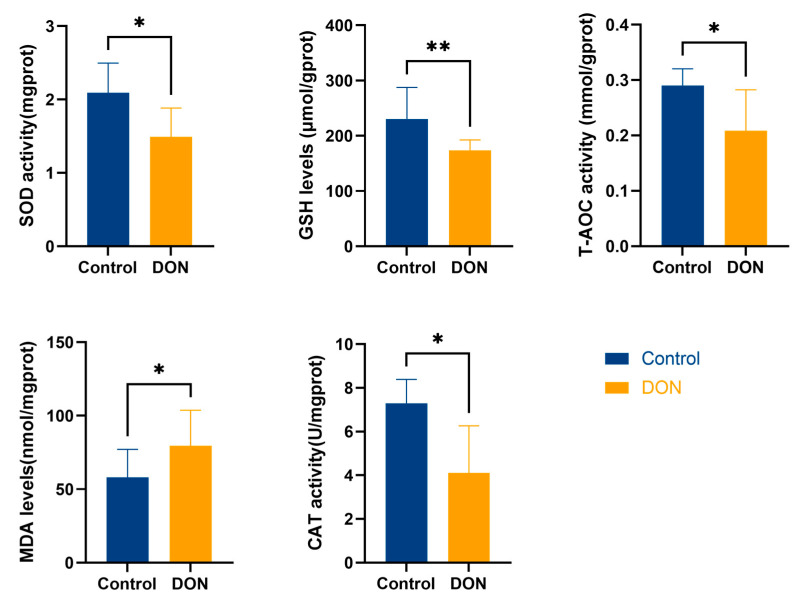
Changes in oxidative stress-related indices in mouse liver. Activities of SOD, GSH, T-AOC, MDA, and CAT in mouse liver cells (*n* = 12, values represent ± SEMs), * (*p* < 0.05) vs. Control, ** (*p* < 0.01) vs. Control.

Oxidative stress is an essential aspect of liver injury caused by DON, so we examined liver oxidative stress-related indices in mice. As shown in Figure 2, the values of SOD, GSH, T-AOC, and CAT were significantly decreased in the DON group compared with the control group (*p* < 0.05), and this result implies that DON exposure causes oxidative stress in the mouse liver. Figure 2, MDA, one of the hallmark indicators of ferroptosis, was significantly elevated in the livers of mice in the DON group compared with the control group, suggesting that DON exposure may cause ferroptosis in the mouse liver.

### 3.3. DON-Induced Ferroptosis Signature Pathway and mRNA Expression Levels of Nrf2 and Its Downstream Pathways in Mouse Livers

**Figure 3:**
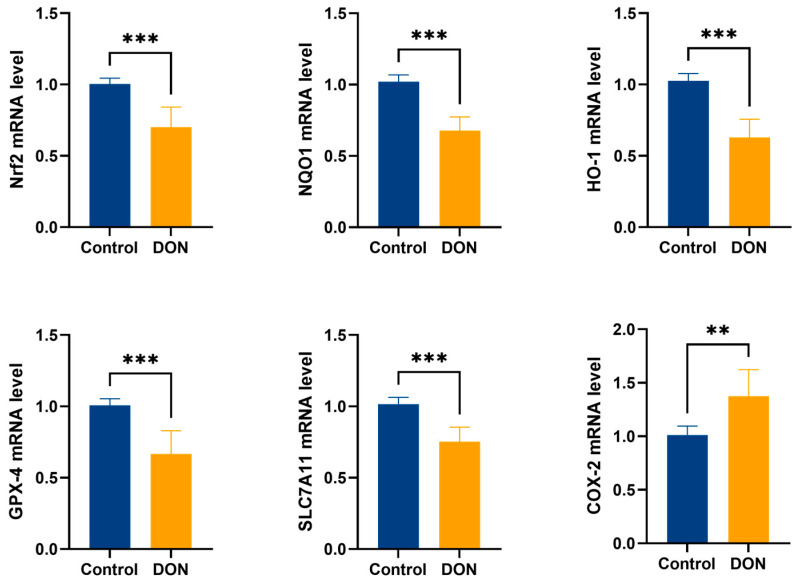
mRNA expression levels of the ferroptosis signature pathway and Nrf2 and its downstream pathways in mouse livers (*n* = 12, values represent ± SEMs), ** (*p <* 0.01) vs. Control, *** (*p* < 0.001) vs. Control.

According to the results in Figure 3, the mRNA expression of COX-2, a signature pathway of ferroptosis, was significantly increased in the DON group compared with the control group (*p* < 0.05), which further demonstrated that DON could induce ferroptosis in mouse livers. Meanwhile, the mRNA expression of Nrf2 and its downstream pathways GPX4, SLC7a11, HO-1, and NQO1 showed a significant decrease under the effect of DON (*p* < 0.01), which indicated that DON induced ferroptosis in the mouse liver by inhibiting the expression of Nrf2 and its downstream pathways.

### 3.4. DON-Induced Ferroptosis Signature Pathway and Protein Expression Levels of Nrf2 and Its Downstream Pathways in Mouse Livers

**Figure 4:**
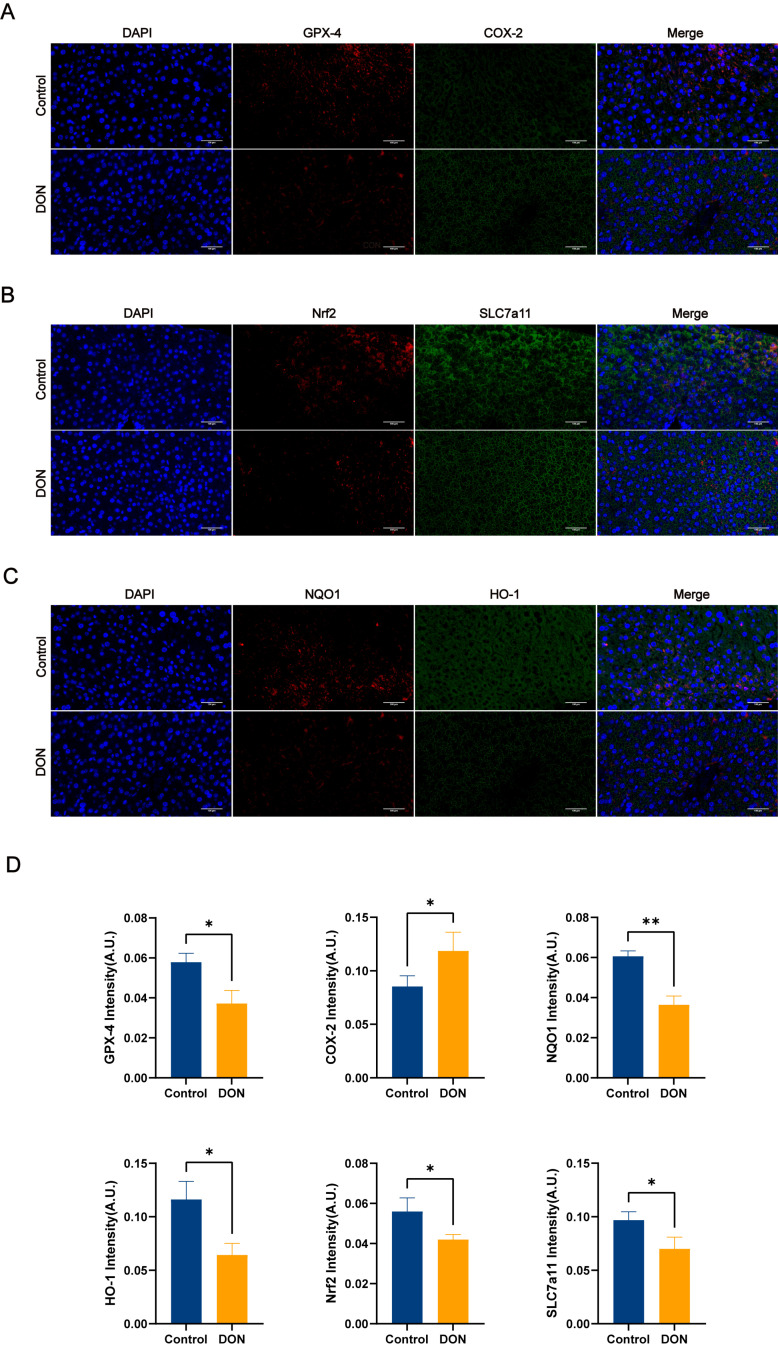
Immunofluorescence double-staining results of the signature pathway of ferroptosis as well as Nrf2 and its downstream pathway in mouse livers (magnification ×10, bar = 100 μm). (**A**) Diagram of double-staining results of GPX4 (red) + COX-2 (green) in mouse livers. (**B**) Double-staining results of mouse liver NQO1 (red) + HO-1 (green). (**C**) Diagram of double-staining results of mouse liver Nrf2 (red) + SLC7a11 (green). (**D**) Immunofluorescence staining fluorescence intensity number values (*n* = 3, values represent ± SEMs), * (*p <* 0.05) vs. Control, ** (*p* < 0.01) vs. Control.

**Figure 5:**
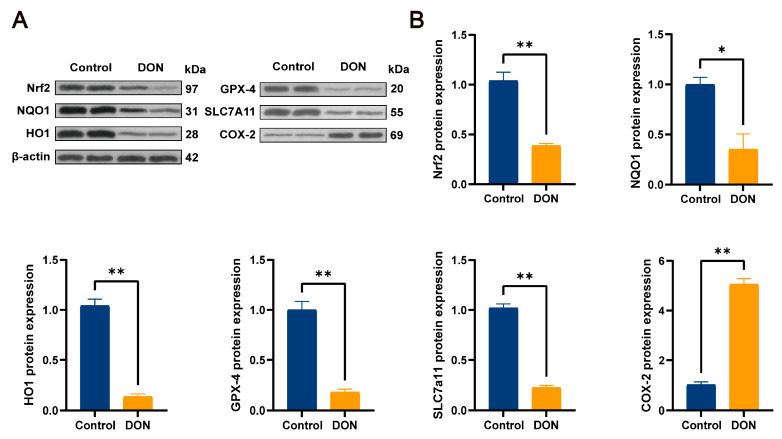
Results of immunoblotting analysis of ferroptosis signature pathways as well as Nrf2 and its downstream pathways in mouse livers. (**A**) Nrf2, NQO1, HO-1, GPX4, SLC7a11, COX-2, and β-actin protein blot bands. (**B**) Nrf2, NQO1, HO-1, GPX4, SLC7a11, and COX-2 protein expression levels (*n* = 3, values represent ± SEMs), * (*p* < 0.05) vs. Control, ** (*p* < 0.01) vs. Control.

In the protein expression of the pathway, we used both immunofluorescence double staining and Western blotting analysis for detection. According to the immunofluorescence double-staining results in Figure 4, the fluorescence intensity of the COX-2 pathway proteins in the control group was lower than that in the DON group (*p* < 0.05), which further proved that DON could induce ferroptosis in mouse livers. Meanwhile, the fluorescence intensity of Nrf2 and its downstream pathways GPX4, SLC7a11, HO-1, and NQO1 in the DON group was significantly lower than that in the control group (*p* < 0.05). The same protein expression trend as the above results was also shown in the Western blotting analysis results in Figure 5, where the COX-2 protein expression level in the control group was significantly lower than that in the DON group (*p* < 0.05), whereas the expression levels of Nrf2 and its downstream pathways GPX4, SLC7a11, HO-1, and NQO1 were significantly higher in the control group than in the DON group (*p* < 0.01). These results suggest that DON induces ferroptosis in mouse livers by inhibiting the expression of Nrf2 and its downstream pathways.

## 4. Discussion

DON contamination has become a common problem in food safety, and it has further become a threat to human and animal health and liver homeostasis [19,20]. This is attributed not only to the high toxicity of DON and its abundance in human and animal food [21] but also to its excellent thermal stability. DON remains stable at temperatures in the range of 170 °C to 350 °C, with its concentration not decreasing even after heating at 170 °C for 30 min [22]. This shows that the high risk of DON should be taken seriously. The liver is the main organ for DON metabolism and is a vital target organ for its toxicity [10]. It has been verified that DON can not only cause severe damage to the liver but also trigger ferroptosis in the liver [14], but the specific pathway and mechanism of DON-induced iron metabolism and ferroptosis in the liver remains unclear. Therefore, in our experiments, we investigated the potential role of the Nrf2 pathway in the process by which DON induces iron metabolism and ferroptosis in the liver of mice.

In this study, the relative weights of the mouse livers were first monitored after sampling, and we found that the organ coefficients of the DON group were significantly higher than those of the control group. This result can be seen to initially determine that DON affected the livers of the mice to a certain extent. Previous studies found that DON could cause inflammation in the liver with specific pathological manifestations such as abnormal hepatic cord and inflammatory cell infiltration [23,24]. Interestingly, in the histopathological analysis of the liver, not only were inflammatory cell infiltration and hepatocyte apoptosis found, but vacuoles produced by hepatocyte steatosis were also found in the liver tissue. Additionally, lipid peroxidation, an essential marker of ferroptosis, also suggests that ferroptosis-associated inflammation in the liver may occur later. To further confirm the effects of DON on the liver, we used mouse serum for liver function-related indexes. Serum ALP, ALT, AST, GLB, and T-bil are essential indicators of liver function and pathology [25]. Specifically, AST and ALT levels are commonly used in assessing hepatic injury, and both are sensitive indicators for assessing the presence of liver damage [26]. In our results, we observed a significant increase in both AST and ALT levels in serum after DON exposure, as well as substantial changes of varying degrees in the other four indicators used to assess the occurrence of liver injury and inflammation, ALB, ALP, GLB, and T-bil. This result, combined with the above histopathological analyses, more conclusively confirms the damage caused by DON to the liver.

Oxidative stress is a key factor in the hepatotoxic effects of DON. During the biotransformation of DON, ROS accumulate in excess and eventually disrupt cell membrane lipids, thus leading to oxidative stress in the liver [6]. In previous studies, it was found that the antioxidant activity of SOD prevents the elimination of reactive free radicals, making it an important antioxidant. GSH converts toxic peroxides into non-toxic hydroxyl compounds, protecting the membrane structure and function. CAT is an early marker of oxidative stress, and its main role is to break down hydrogen peroxide, thus maintaining redox balance in the cell. At the same time, T-AOC represents the total antioxidant level in the organism, encompassing a variety of antioxidants and antioxidant enzymes, among others, because these antioxidant substances and antioxidant enzymes protect cells and organisms from oxidative stress damage caused by reactive oxygen radicals [27]. In our results, the levels of GSH, CAT, SOD, and T-AOC were significantly decreased in the DON group compared with the control group, suggesting that DON enhances ROS formation and reduces the total antioxidant level in mouse livers, leading to oxidative stress. Meanwhile, excessive ROS may trigger lipid peroxidation and impair cellular macromolecule function. MDA is a biomarker of lipid peroxidation, which is also a golden indicator for the occurrence of ferroptosis [28]. In the present study, we observed that DON significantly increased MDA levels in the liver. This not only represents that DON triggers the occurrence of lipid peroxidation in the liver but also further suggests the occurrence of ferroptosis.

Ferroptosis is a novel type of cell death characterized by iron-dependent lipid peroxidation, oxidative stress, and iron overaccumulation. The above experimental data have confirmed that DON can trigger oxidative stress and hepatic lipid peroxidation. To further determine whether ferroptosis occurs, we found that DON could increase Fe^2+^ content in the liver tissues of mice after examining the Fe^2+^ content in the liver. MDA and Fe^2+^ levels have been seen to be characteristic and key indicators of ferroptosis, but as of this writing, the definitive mechanism of the link between lipid peroxidation and ferroptosis is not entirely clear. Although MDA and Fe^2+^ levels cannot be regarded as absolute indicators of ferroptosis, they can still serve as an essential basis for determining whether ferroptosis occurs or not as far as the current situation is concerned. In the following study, we will endeavor to explore more scientifically sound indicators as a measure. Therefore, based on the above results, we have a strong basis to conclude that DON induces pyroptosis in the liver.

Nrf2 is a key factor in the regulation of apoptosis. A previous study found that ferroptosis inducers can initiate a series of ferroptosis programs by inhibiting GPX4 and SLC7a11, both downstream targets of Nrf2 [13]. GPX4 is a member of the glutathione peroxidase family that catalyzes the reduction of lipid peroxides, thus preventing lipid peroxidation-induced damage to cell membranes. GPX4 also plays a key role in ferroptosis, and targeted intervention in GPX4 expression can inhibit the occurrence of ferroptosis [29]. SLC7a11, on the other hand, is a component of system Xc and is responsible for cystine uptake, thereby promoting the synthesis of GSH. Whereas GSH is a substrate for GPX4, SLC7a11 can inhibit ferroptosis occurrence by promoting GSH synthesis to provide a substrate for GPX4 [30,31]. This observation is consistent with the results obtained in our experiments. Our data showed that the mRNA and protein expression levels of Nrf2, SLC7a11, and GPX4 genes were significantly lower in the DON group than in the control group. This suggests that DON may inhibit SLC7a11 expression by regulating Nrf2, thereby reducing GSH synthesis. Eventually, GSH depletion and GPX4 inactivation promoted the increase in lipid peroxides in the hepatocytes, which led to ferroptosis. Apart from that, Nrf2 also plays a crucial role in antioxidants [32,33], and oxidative stress is one of the hallmarks of ferroptosis. Nrf2 regulates the expression of several antioxidant genes, including antioxidant enzymes (GPX1, GPX4, SOD1, and CAT) and phase II antioxidant enzymes (HO-1 and NQO1), thereby attenuating tissue damage and, during oxidative stress, maintaining a normal redox state [34,35]. In addition, in a previous study, it was found that intracellular iron metabolism could be regulated by up-regulating the expression levels of Nrf2, HO-1, and NQO1, preventing iron overaccumulation and lipid peroxidation, and thus, alleviating radiation-induced pulmonary fibrosis [36]. So, in the experiment, we examined the mRNA and protein expression levels of HO-1 and NQO1. The assay results showed that DON substantially reduced the mRNA and protein expression levels of the HO-1 and NQO1 genes. This suggests that DON may down-regulate the expression of HO-1 and NQO1 genes via Nrf2 to exacerbate oxidative stress and iron overaccumulation in the liver, resulting in ferroptosis. This echoes our findings of oxidative stress indicators and Fe^2+^ content above. Interestingly, ferroptosis is also closely linked to the key inflammatory mediator COX-2. Typically, COX-2 can regulate inflammation and cell proliferation by synthesizing prostaglandins (PGs) and thromboxanes from arachidonic acid (AA) [37]. Further investigation found that ferroptosis directly increases the expression of prostaglandin–endoperoxide synthase 2 (PTGS2)-encoding COX-2, which accelerates AA metabolism and promotes the secretion of inflammatory signaling molecules [38]. Recently, a new study also found that hyperoxia-activated Nrf2 regulates ferroptosis in intestinal epithelial cells and intervenes in inflammation through the COX-2/PGE2/EP2 pathway [39]. In the pathological analysis results above, we found the presence of inflammatory cell infiltration in hepatocytes in the DON group. Therefore, it is reasonable to speculate that DON can also regulate the COX-2 pathway through Nrf2 to cause inflammation in the liver, thus inducing ferroptosis. The results of our COX-2 gene mRNA and protein expression levels showed a clear trend of increasing COX-2 gene mRNA and protein expression levels in the DON group compared with the control group. This may be consistent with our speculation. However, since the primary focus of this experiment was not to investigate the relationship between DON-induced liver inflammation and ferroptosis, this result remains only speculation. Notably, the finding of this result further implies that DON-induced liver inflammation is likely to have a non-negligible relationship with ferroptosis.

While many scholars have explored the close association between COX-2 and DON-induced inflammation, the relationship between COX-2 and ferroptosis remains unexplored. In our future research, it will be fruitful to conduct in-depth understanding and experiments in this direction and strive to obtain more sufficient data to confirm this conjecture continuously.

In the present study, the damage caused by DON to the mouse liver was corroborated by histological analysis of the liver, relative liver weight, and changes in liver function indices. It was further confirmed that DON induced hepatic ferroptosis by detecting hepatic Fe^2+^ and MDA content. To investigate the link between Nrf2 and DON-induced ferroptosis in the liver, we examined and analyzed the expression levels of downstream genes related to Nrf2 in terms of oxidative stress and lipid peroxidation. As a relatively new concept of death, ferroptosis cuts across several essential aspects of iron metabolism, oxidative stress, lipid peroxidation, and inflammation. While our study has led to a better understanding of the mechanism of DON-induced ferroptosis in mouse livers, it primarily focused on elucidating the changes in gene expression. The exact mechanism by which Nrf2 and downstream pathways regulate DON-induced ferroptosis remains elusive. In this regard, further investigation is needed to clarify the precise mechanism. In the first exploration of the link between the Nrf2 pathway and DON-induced ferroptosis in mouse livers, this finding could contribute to the future development of potentially more effective DON-targeted antidotes.

## 5. Conclusions

In summary, DON can induce ferroptosis in mouse livers by regulating the Nrf2 pathway and its downstream genes, thus causing hepatotoxicity. The main manifestations were oxidative stress injury, lipid peroxidation injury, and increased Fe^2+^ content in the liver. The liver is a vital target organ for the accumulation of toxins in the body, and the damage caused by DON to the liver should not be ignored. This study provides accurate and reliable evidence as well as new perspectives, suggesting that Nrf2 may be a potential target for mitigating DON hepatotoxicity in the future.

## Data Availability

The data presented in this study are available on request from the corresponding author.

## Supplementary Materials

The following supporting information can be downloaded at: https://www.mdpi.com/article/10.3390/metabo15030165/s1, Figures S1–S7: Immunoblot complete bands; Table S1: Primers used for quantitative real-time PCR.

## Abbreviations

The following abbreviations are used in this manuscript:

DON	Deoxynivalenol
GSH	Glutathione
CAT	Catalase
SOD	Superoxide dismutase
T-AOC	Total antioxidant capacity
MDA	Malondialdehyde
HO-1	Heme oxygenase
NQO1	Quinone oxidoreductase 1
GPX4	Glutathione peroxidase 4
SLC7a11	Solute Carrier Family 7 Member 11
DON-GlcA	DON-glucuronide
UGT	UDP-Glycosyltransferase
HE	Hematoxylin and eosin
PG	Prostaglandins
AA	Acid
PTGS2	Prostaglandin–endoperoxide synthase 2
