# Supplementary material for: Disorders of Iron Metabolism: A “Sharp Edge” of Deoxynivalenol-Induced Hepatotoxicity
Source: Metabolites. 2025 Mar 1;15(3):165. doi: 10.3390/metabo15030165 (PMC11943501; doi:10.3390/metabo15030165)
Supplement: Supplementary file 1 [file metabolites-15-00165-s001.zip › metabolites-3462413-supplementary.pdf]

Supplementary Material

1 Supplementary Data

Figure S1-S7. Immunoblot complete bandsTable

Table S1. Primers used for quantitative real-time PCR

2 Supplementary Figures and Tables

2.1 Supplementary Figures

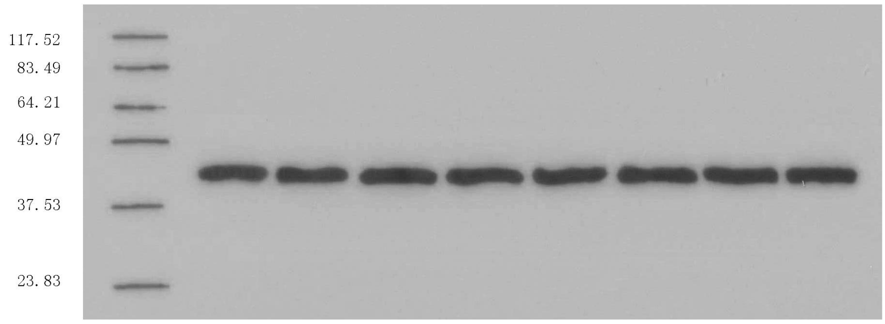

Figure S1. β-actin immunoblot complete bandsTable

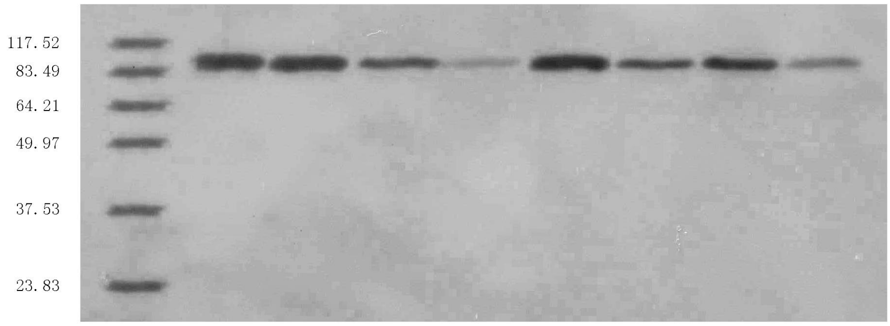

Figure S2. Nrf2 Immunoblot complete bandsTable

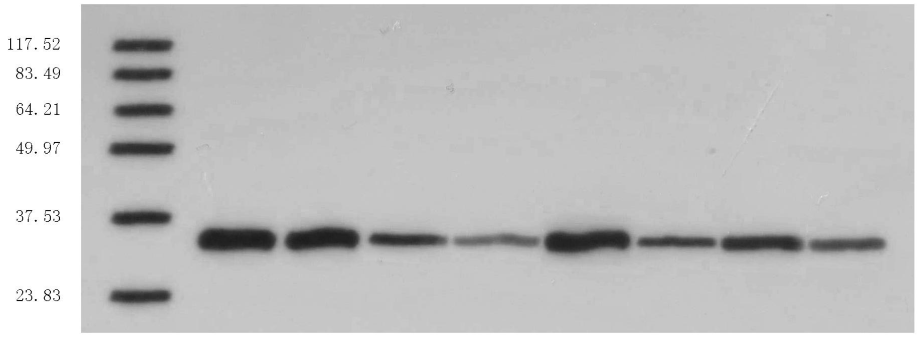

Figure S3. NQO1 immunoblot complete bandsTable

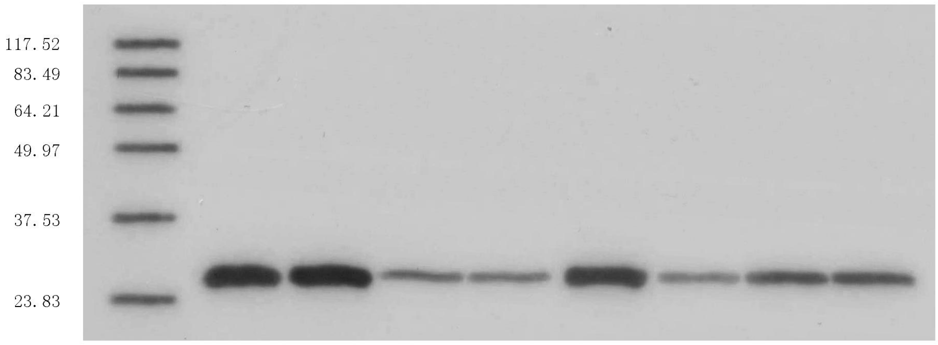

Figure S4. HO-1 immunoblot complete bandsTable

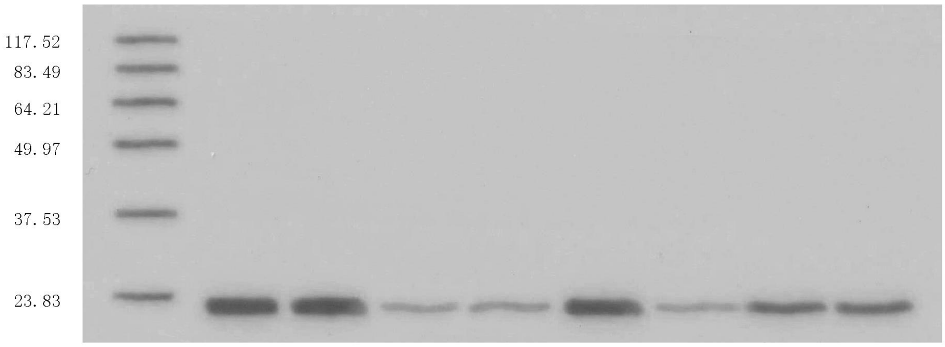

Figure S5.GPX-4 immunoblot complete bandsTable

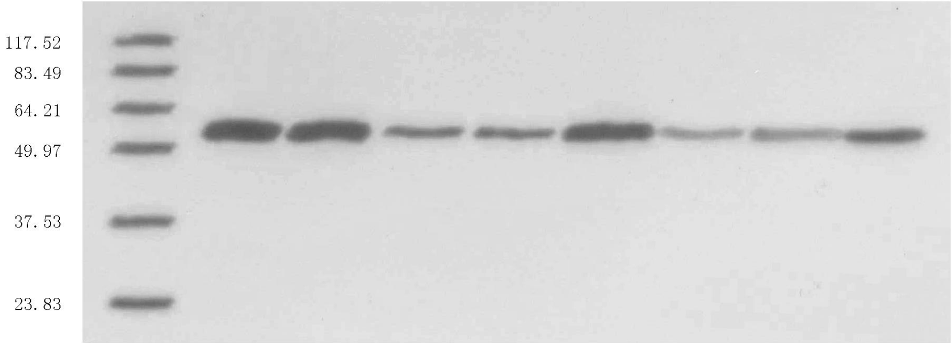

Figure S6.SLC7a11 immunoblot complete bandsTable

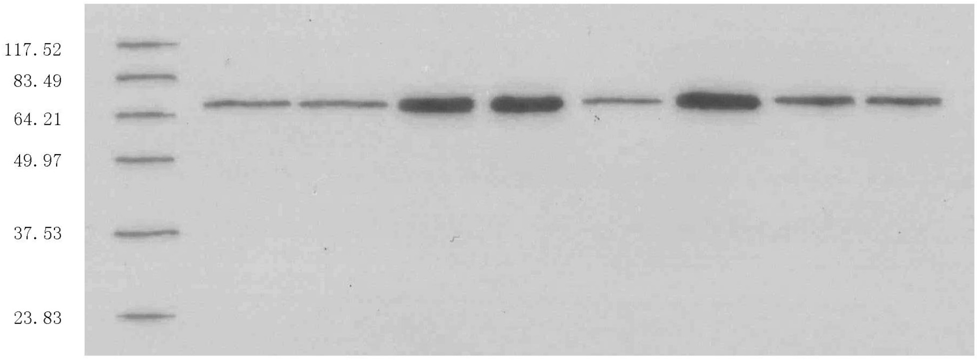

Figure S7.COX-2 immunoblot complete bandsTable

2.2 Supplementary Table

Table S1. Primers used for quantitative real-time PCR

Primer Name	Accession No.	Forward Primer （5’→ 3’）	Reverse Primer （5’→ 3’）
β-actin	NM_001289726.2	CGACTTCAACAGCAACTCCCACTCTTCC	TGGGTGGTCCAGGGTTTCTTACTCCTT
Nrf2	NM_001399226.1	TATCTCCTAGTTCTCCGCTGCTC	GTGGCAACTCCAAGTCCATCAT
HO-1	NM_010442.2	ACCGCCTTCCTGCTCAACATTG	CTCTGACGAAGTGACGCCATCTG
NQO1	NM_001204272.2	ATCCTGCGTTTCTGTGGCTTCC	TCCTCCCAGACGGTTTCCAGAC
GPX-4	NM_001037741.4	GCCTGGATAAGTACAGGGGTT	CATGCAGATCGACTAGCTGAG
SLC7a11	NM_011990.2	ACCACCATCAGTGCGGAGGAG	ATGGAGCCGAAGCAGGAGAGG
COX-2	NM_033292.4	AGACGAAATCAACAACCCCGT	AGCAGTCGTAGTTCACCAGG
